# Cell-Based Transplantation versus Cell Homing Approaches for Pulp-Dentin Complex Regeneration

**DOI:** 10.1155/2021/8483668

**Published:** 2021-09-29

**Authors:** Geraldine M. Ahmed, Eman A. Abouauf, Nermeen AbuBakr, Asmaa M. Fouad, Christof E. Dörfer, Karim M. Fawzy El-Sayed

**Affiliations:** ^1^Stem Cell and Tissue Engineering Research Group, Faculty of Dentistry, Cairo University, Cairo, Egypt; ^2^Department of Endodontics, Faculty of Dentistry, Cairo University, Cairo, Egypt; ^3^Department of Conservative Dentistry, Faculty of Dentistry, Cairo University, Cairo, Egypt; ^4^Oral Biology Department, Faculty of Dentistry, Cairo University, Cairo, Egypt; ^5^Clinic for Conservative Dentistry and Periodontology, School of Dental Medicine, Christian Albrechts University, Kiel, Germany; ^6^Oral Medicine and Periodontology Department, Faculty of Dentistry, Cairo University, Cairo, Egypt

## Abstract

Regenerative dentistry has paved the way for a new era for the replacement of damaged dental tissues. Whether the causative factor is dental caries, trauma, or chemical insult, the loss of the pulp vitality constitutes one of the major health problems worldwide. Two regenerative therapies were introduced for a fully functional pulp-dentin complex regeneration, namely, cell-based (cell transplantation) and cell homing (through revascularization or homing by injection of stem cells in situ or intravenously) therapies, with each demonstrating advantages as well as drawbacks, especially in clinical application. The present review is aimed at elaborating on these two techniques in the treatment of irreversibly inflamed or necrotic pulp, which is aimed at regenerating a fully functional pulp-dentin complex.

## 1. Introduction

Dental tissue regeneration requires the presence of specialized cells capable of the production of a tissue-specific extracellular matrix (ECM) [1, 2]. Stem/progenitor cells used in regenerative medicine are nonspecialized cells, demonstrating the ability of self-renewal and multilineage differentiation [3]. The potential of stem/progenitor cells, whether endogenous or exogenous, to adapt to various environmental niche could be exploited in regenerative endodontics and pulp-dentin tissue regeneration [4–6]. Therapeutic application of stem/progenitor cells is mainly dependent on the utilization of the transplanted cells, on suitable scaffolds and in combination with various growth factors to generate fully functional biological tissues [7]. Recently, the success demonstrated in animal models to repair/regenerate dental structures has paved the way for pulp-dentin organ regeneration approaches [8].

### 1.1. Cell-Based Transplantation for Pulp-Dentin Complex Regeneration (Table 1 and Figure 1)

A suggested approach to address problems related to pulp-dentin tissue regeneration relied principally on the use of various sources of stem/progenitor cells, combined with multiple scaffold systems and growth factors [9]. Human mesenchymal stem/progenitor cells (MSCs) have been extracted from many areas of the human body, including the bone marrow, the skin as well as the perivascular, the adipose, and the dental tissues [10–12]. Early trials and continuous animal studies were directed to investigate the effectiveness of cell-based transplantation on pulp healing and dentin regeneration [7, 13, 14]. Autologous transplanted constructs of dental pulp stem/progenitor cells (DPSCs) in combination with platelet-rich fibrin (PRF) proved to promote the regeneration of pulp-dentin-like tissue inside dogs' root canals [15]. A further animal study employing human DPSCs and platelet-derived growth factor (PDGF) constructs transplanted into the emptied root canals of rats induced the creation of globular dentin-like structure with odontoblastic cells and pulp-like tissues [16].

A trial to treat deliberately perforated pulp space of premolars of dogs using autogenous DPSCs, embedded in tricalcium phosphate (TCP) or treated dentin matrix (TDM) scaffolds, showed no dentin formation in all groups while cementum and vascular connective tissues were evident in all specimens [17]. A further study examined microvascular endothelial cells (ECs) coimplanted with rat bone marrow MSCs in pulpotomized rat models. Interestingly, after 14 days, immunohistochemical examination demonstrated healing of the pulp with complete dentin bridge formation in teeth implanted with MSCs and ECs, while those implanted with MSCs lacked the completion of the formed dentin bridge [18]. A further noninvasive regenerative pulpal approach was tested, using mobilized DPSCs freshly extracted from upper canine teeth of dogs, followed by autologous DPSCs transplantation in pulpectomized permanent teeth with apical closure. The study revealed that pulp tissue was completely regenerated 90 days following cell transplantation [19]. A novel trial on a rat model for dental pulp regeneration employed pulpotomized rat teeth, which were treated using buildups of rat bone marrow mesenchymal stem cells (BMMSCs). The tested buildups were implanted into the pulpotomized pulp chambers for 3, 7, or 14 days and then examined immunohistochemically. At 7 days, the pulp tissue was regenerated in almost the whole implantation area and regeneration continued to progress for 14 days with differentiation of odontoblast-like cells beneath the dentin at the margin of the implanted area evidenced by a detected nestin expression. Also, quantitative gene expression analysis disclosed the expression of sialophosphoprotein mRNA in the implanted area, suggesting the abundance of odontoblasts [20]. Chitosan hydrogel scaffold containing autologous DPSCs was further transplanted in the necrotic immature permanent teeth of dogs, regenerating pulp- and dentin-like tissues with complete root maturation radiographically and histologically [21]. However, not all the reported studies were successful. Implanting DPCs in TCP and TDM scaffolds, combined with transforming growth factor *β*, ascorbic acid 2-phosphate, and ascorbic acid 3-phosphate, did not promote the formation of a dentin bridge [17]. Also, porcine DPCs failed to heal or regenerate partial pulpotomy defects of minipigs. Moreover, hyperemia in the residual pulp and external root resorptions were evident in the radicular area of all the treated teeth [22]. On the contrary, another investigation demonstrates that when combining collagen scaffold with granulocyte colony-stimulating factor (G-CSF), a total recovery of the pulp tissue was achievable in the pulpectomized teeth [19].

It was appealing to seek more uncommon supplementary derivatives to enhance stem/progenitor cells' activation and differentiation, dragging attention towards nondental medications. An animal study reported that a common drug used to treat hyperlipidemia, Simvastatin (SIM), succeeded in stimulating canine DPSCs, promoting pulp and dentin regeneration following pulpotomy [23]. Further animal studies suggested using glycogen synthase kinase (GSK-3) antagonists, a drug usually applied for the treatment of neurological disorders, which proved successful as a capping material of the pulpal exposure site, promoting dentin formation [24, 25]. Another animal study proved that pulp regeneration was enhanced in aged dogs' teeth by trypsin pretreatment of allogenically transplanted mobilized DPSCs [26].

A case report treating accidental root perforation of a mature permanent tooth, utilizing allogenic umbilical cord mesenchymal stem cells (UCMSCs) encapsulated in a platelet-poor plasma- (PPP-) based bio scaffold, demonstrated a clinically normal pulpal tissue in terms of vitality testing, palpation, and percussion testing at six-month and one-year follow-ups [27]. Moreover, two case reports showed a successful management of periapical lesions in permanent teeth treated with stem/progenitor cells from human exfoliated deciduous teeth (SHED), with the treated teeth responding normally to electric pulp testing and periapical tissue healing observed and maintained up to one year [28].

Collectively, cell-based therapeutic applications in the dental field and specifically dentin-pulp tissue regeneration still face a number of challenges. Future strategies should be directed towards overcoming these challenges and obstacles using an ideal combination of growth factors with properly matching scaffolds [17, 22]. Secure and controllable practice must be strictly followed to translate stem/progenitor cell research into human models, starting from protocols of stem/progenitor cells' tissue harvesting, the biocompatibility of the used scaffolds and biomaterials involved, and the safety of the technique itself and the predicted outcome [29, 30]. Finally, the endless mix and match trials between scaffolds of different origins, as well as electing the suitable growth factor/biological mediator, could govern the success or failure of regenerating a specialized tissue when employing the stem cell-based therapy [31].

### 1.2. Stem/Progenitor Cell Homing

As mentioned above for pulp-dentin complex regeneration, two strategies could be applied, namely, the cell-based transplantation therapy or the cell homing. In the latter, the regeneration is accomplished via chemotaxis of host endogenous cells to the injured tissue via biological signaling molecules. Stem/progenitor cell homing can be defined as the potential of stem/progenitor cells, whether endogenous or exogenous, to migrate into an environmental niche. MSCs can be delivered in situ or intravenously, or they can be recruited to sites of injury, through migration and homing [32]. Clinically, cell homing for pulp-dentin complex regeneration might be simpler and more economical to perform compared to the cell-based therapy and readily performed by clinicians without special training.

### 1.3. Stem/Progenitor Cell Homing Mechanisms (Figure 2)

Homing approaches can be either systemic or nonsystemic. In nonsystemic homing, MSCs are locally transplanted at the selected tissue and are then directed to the region of injury through a chemokine gradient. Oppositely, in systemic homing, MSCs are delivered or recruited endogenously into the circulation and then undergo a series of processes, leaving the bloodstream and moving towards the site of injury. These complex processes involve tethering and rolling, activation, arrest, transmigration or diapedesis, and migration [33, 34]. Tethering is mediated by selectins on endothelial cells. MSCs exhibit CD44, which binds to the selectins and starts rolling along blood vessels [35]. This is followed by chemokine-mediated activation [36]. MSCs express the chemokine receptors CXCR4 [37] and CXCR7 [38, 39]. The stromal cell-derived factor (SDF-1) is the ligand to these receptors, where it binds to them to enhance homing to different tissues. Then, comes the process of arrest mediated by integrins, mainly by CD49d (*α*4*β*1), which unites with VCAM-1 (CD106) present on endothelial cells [40]. In order to cut across the endothelial basement membrane, a process known as diapedesis or transmigration, MSCs produce matrix metalloproteinases (MMPs) mainly MMP-1, which plays a crucial role in tissue infiltration by MSCs [41]. Finally, MSCs migrate to injury sites. This step is regulated by chemotactic signals, produced as a reaction to tissue impairment. Numerous growth factors, such as insulin-like growth factor IGF-1 and platelet-derived growth factor (PDGF), can act as chemokines for MSCs [42]. Moreover, tumor necrosis factor (TNF-*α*) increases MSCs movement towards chemokines by increasing their expression of CCR3, CCR4, and CCR2 receptors [4, 43, 44]. In addition, the inflammatory cytokine interleukin- (IL-) 8 was proved to enhance migration of MSCs towards regions of injury [45, 46] and further promotes them to produce regenerative growth factors, such as vascular endothelial growth factor (VEGF) [47].

### 1.4. Routes of Administration and Delivery Methods

One important point in MSCs transplantation and their consequent therapeutic efficiency is the route of administration to provide the ultimate regenerative benefits with the least adverse effects. The most common delivery methods for MSCs are either by intravenous (IV) or intra-arterial infusion (IA) or by direct intratissue injection [48]. Several experimental studies proved the superiority of IA and IV delivery modes over other delivery routes [49, 50]. The IV route was proved to be the most convenient route for MSCs transplantation. It is less traumatic and reproducible and enhances widespread distribution in the affected regions, enhancing various biological effects [51]. However, this delivery method in nearly all cases causes entrapment of MSCs in the lungs, causing undesirable adverse effects, including embolisms. The reason for this lung entrapment relies probably on the amalgamation of physiological and mechanical factors, such as the small size of blood capillaries, the vast network of capillaries, and the great adhesive characteristics of MSCs. It was also demonstrated that some cells could produce calcium deposits within the capillaries [52].

On the contrary, the IA route can be more efficient, as it provides a straightforward route to the injury site with an increased degree of cellular endurance and engraftment [53, 54]. Several studies proved the superiority of IA delivery route over the IV one. They demonstrated enhanced functional and histological results in IA delivery compared with IV injection of MSCs [49, 55]. IA transplantation of MSCs increases cellular migration, cellular density, and the number of homing MSCs to the target tissue, when compared to IV injection [56, 57]. Du et al. in a comparative study demonstrated greater angiogenesis and increased functional recovery with IA transplantation compared to IV injection utilizing human BM-MSCs in a rat model of ischemia [58]. Lundberg et al. confirmed these findings in a model of traumatic brain injury [59]. The main reason for the superiority of IA transport over IV mode is that the IA approach can bypass the pulmonary circulation and filtering organs, such as liver and spleen [60], thereby avoiding MSCs entrapment in lungs and liver [54], with a significant rise in number of cells with a more consistent cellular dissemination in target tissues [61, 62]. This will eventually lead to increased cell homing and improved therapeutic outcomes [58].

However, a probable limitation for the IA route is the possibility of vascular blockage in small arterioles and capillaries resulting in strokes. This may be attributed to the existence of large MSCs in the 20–50 *μ* size range [63, 64]. Several attempts have been performed to enhance the safety of IA transplantation via regulating infusion velocity and cell dosage [63, 65]. Moreover, real-time MRI could provide a useful tool in making the procedure more accurate and predictable, which is of ultimate importance for translation to clinical practice [66].

Direct injection delivery mode has the advantage of accurate localization of cells, despite being invasive. However, it has been proved that aside from the delivery route, only 1∼5% of delivered cells disseminate within the target region for regeneration. The count of cells in the target region may thus be enhanced by maximizing the injection volume or enriching the cell concentration in the injectable volume [67–69]. In addition, the expression of adhesion molecules can promote homing of delivered MSCs [70, 71]. In this context, several approaches have been made to enhance MSC homing efficacy.

### 1.5. Enhancing MSC Homing (Figure 2)

Cellular homing relies principally on specialized molecular interactions, not just passive diffusion. One of the main challenges facing MSCs therapeutic applications is enhancing their homing abilities [72]. Among the challenges is the fact that the expression of homing molecules, as CXCR4, is relatively low on MSCs [37, 73], and the in vitro expansion of MSCs further decreases the expression of their homing molecules [74, 75]. Thus, numerous approaches have been suggested to enhance MSC homing. Among these is targeted delivery, which relies on direct delivery of MSCs into the target region, employing nonsystemic rather than systemic homing [76]. In addition, magnetic guidance of MSCs to target tissues proved greater homing efficiency [77]. Furthermore, genetic modifications of MSCs via overexpression of homing factors such as VLA-4 and CXCR4 through viral transduction proved increased efficiency [78, 79]. Cell surface engineering approaches were suggested to modify the selectin ligand CD44, via transforming it into HCELL (the ligand for E- and L-selectin that MSCs utilize for homing), as MSCs normally express CD44, but not HCELL [80]. It was further demonstrated that coating MSCs with hyaluronic acid could upregulate CD44 expression [81]. Moreover, hypoxic conditions enhance hypoxia-inducible factor- (HIF-) 1a, which upregulates the expression of CXCR4 [82], CX3CR1 [83], and CXCR7 [84, 85].

A further strategy addressed modifying the target tissues, via overexpression of chemokines or via implantation of chemokine-coated scaffolds [86]. This allows tissues to be a more appealing target for homing MSCs. Moreover, irradiation of target tissues increases the expression of SDF-1, upregulating in MSC engraftment [87, 88] and homing [89]. Pulsed ultrasound applied to the target tissue may also enhance MSC homing [90], via altering gene expression of cytokines as bone morphogenic protein-2 (BMP-2), interleukins (IL-1*α*, IL-6, and IL-10), TNF-*α*, and growth factors such as epidermal growth factor (EGF), fibroblast growth factor (FGF), VEGF, and PDGF [91], causing disorganization of endothelial linings, enhancing vascular permeability, increasing secretion of SDF-1 on the tissue of interest, and upregulating CXCR4 expression [92].

### 1.6. Cell Homing for Pulp-Dentin Complex Regeneration (Revascularization) (Table 2 and Figure 3)

Regenerative endodontics represents an alternative to root canal treatment, which is aimed at replacing the inflamed and necrotic pulp tissue with regenerated pulp-like tissue [93]. In this context, revascularization approaches of affected dental pulp were suggested as an innovative strategy to overcome the drawbacks associated with classical root canal treatment methods (e.g., fracture of the teeth and loss of vitality) [94]. A human study on mature necrotic teeth with large radiolucency concluded that the regenerative endodontic approaches have a success rate similar to nonsurgical endodontic treatment as a therapeutic alternative for mature necrotic teeth with radiolucency [95]. It could maintain the pulp vitality, leading to a reduction of apical periodontitis and enhance the periapical healing mechanism [96]. Basically, pulp revascularization is the reestablishment of angiogenesis inside the root canal but without the repopulation of odontoblasts, while the pulp regeneration means angiogenesis with presence of odontoblastic layer lining the dentinal surface, nociceptive as well as parasympathetic and sympathetic nerve fibers, interstitial fibroblasts, and stem/progenitor cells, which replenish the pulp cells in the newly regenerated pulp tissue [97]. According to American Association of Endodontists' (AAE) Clinical Considerations for a Regenerative Procedure, the primary goal should be the resolution of clinical symptoms/signs and elimination of apical periodontitis. The secondary goal should address the canal wall thickening and/or continued root maturation [98].

Pulp revascularization could be considered a type of cell homing strategy for pulp-dentin complex regeneration. This clinical procedure depends on the delivery of a blood clot (scaffold) inside the root canal, growth factors (mainly from platelets and dentin), and stem/progenitor cells. The stem/progenitor cells of interest in revascularization are SCAP (stem cells of apical papilla) because of their anatomical positioning immediately adjacent to the termination of the root canal system, permitting easy cell delivery to the root canal [99, 100] and the greater superiority for dentin-like tissue formation [101, 102]. The root canal system is first disinfected with a combination of antibiotics or calcium hydroxide. In the second visit, the irrigation protocol during this clinical procedure is very critical as for the regeneration procedure to be successful; the irrigants should have bactericidal/bacteriostatic properties as well as an ability to promote survival and proliferative capacity of the patient's stem/progenitor cells. The irrigation protocols that include 17% EDTA promoted SCAP survival and attachment to the root canal dentinal wall [103].

Animal studies were performed to examine the tissues formed after revascularization, demonstrating ingrowth of cellular cementum-like tissues, formation of pulp-like tissue, thickening of the canal walls, closure of the root apex, and disappearance of periapical radiolucency [104, 105]. Histological sections were also performed in humans after fracture of a revascularized immature tooth (3.5 weeks after revascularization), showing that the canal was filled with loose connective tissue and a layer of flattened odontoblast-like cells lined along the predentin. Layers of epithelial-like cells, similar to the Hertwig's epithelial root sheath, further surrounded the root apex [106].

Alternative endodontic therapy is now possible, using the patient's own blood samples, where PRF and PRP are introduced inside the root canal. Easier and successful efforts for pulp revascularization and pulp tissue regeneration were reported by using evoked bleeding (EB), where the blood clot acts as a protein scaffold and interacts with endogenous stem cells and growth factors already abundant in the adjacent bone marrow tissues [107]. The highest reported cytokines and growth factors found in PRF are IL-1*β*, IL-6, IL-4, TNF-*α*, PDGF, VEGF, IGF-1, EGF, and transforming growth factor *β*1 (TGF*β*1) [108], while PRP contains FGF, PDGF, VEGF, IGF-1, EGF, and TGF*β*1 [109]. The superiority of PRP came from releasing an elevated number of proteins at early time intervals whereas PRF showed a sustained production of bioactive molecules throughout a duration of 10 days [110]. In the blood clot technique, the growth factors are released from the dentin matrix after conditioning of the dentin using EDTA (ethylene diamine tetra acetic acid) 17%-pH 7.2 during the revascularization technique. Thus, the dentin matrix acts as a reservoir of bioactive molecules, which provides a vital source of cell signaling molecules for initiating repair, including TGF*β*1, bone morphogenetic proteins (BMPs), and VEGF [111]. PRF has proved to be an appropriate substitute to the blood clot technique, especially in cases where bleeding was very difficult to be obtained [107]. PRP and blood clotting technique used as scaffolds in immature traumatized permanent teeth with necrotic pulps also gave very good results [112]. In a clinical study on 30 patients with maxillary necrotic permanent immature central incisors, treating one group with PRP and the other with PRF scaffolds, teeth survived during the 12-month follow-up period. The teeth revealed marginal increase in radiographic root width and length, an increased periapical bone density, and a narrowing in apical diameter [113]. Other studies compared the effect of PRF, PRP, and the blood clot technique in the revascularization of necrotic teeth with open apex, demonstrating continued root development and maintenance of functionality, following different follow-up periods, yet with some teeth not responding to vital testing [2, 5, 6, 114–122]. A further investigation induced bleeding in root canals and used PRF in mature necrotic teeth, showing a regain in pulp sensibility [123]. In a further study, Kim et al. were able to regenerate tooth-like structure using cell homing approach [124].

Still, one of the drawbacks of the revascularization found among cases treated with this approach is the occasional intracanal calcification, which in some cases may progress to complete obliteration of root canals, affecting the normal function of the dental pulp tissues. This drawback could be attributed to multiple contributing factors such as the type of medicaments and the induction of intracanal bleeding [125, 126]. A recent review article evaluated the long-term outcomes of the apexification and the regenerative techniques in treating traumatized immature teeth with pulp necrosis and apical periodontitis, showing that the endodontic regenerative techniques appeared superior to apexification techniques in terms of root lengthening and root wall thickening [127].

### 1.7. Cell-Free Approach for Pulp-Dentin Complex Regeneration

Relying on “cell homing” concept, the cell-free approach is aimed at regeneration by enhancing proliferation, migration, and differentiation of intuitive stem/progenitor cells present near the root apex [128]. It was proposed that stem/progenitor cells' niches could initiate an appropriate microenvironment by releasing immunoregulatory molecules and enhancing paracrine effects to promote the differentiation of endogenous stem cells [129, 130]. Additionally, natural molecules and bioactive compounds have been proved to promote dentinogenesis [131, 132].

Conditioned medium (CM) can be described as the molecules released from living cells into the surrounding extracellular environment [133]. CM was found to stimulate cellular immunomodulation, proliferation, migration, and tissue regeneration [133–135] as it contains abundant amounts of proteins, lipids, nucleic acid, growth factors, cytokines, chemokines, and extracellular vesicles [136]. A recent study combined hDPSC conditioned medium with MTA for direct vital pulp therapy. It was assumed that the abundance of angiogenic growth factors such as PDGF, FGF, and VEGF [137] and immunomodulatory cytokines such as IL-6 and IL-8 [138] secreted by DPSCs and collected in hDPSCs' conditioned medium could modulate the inflammatory and regenerative processes in the dental pulp tissue, improve the orientation of the newly formed hard tissue, and enhance formation of dentin bridges [139].

Extracellular vesicles (EVs) derived from MSCs function as paracrine mediators in tissue regeneration and repair and resemble to a great extent the therapeutic efficacy of parental MSCs [140]. Extracellular vesicles (EVs) are defined by the MISEV2014 and the updated MISEV2018 as “particles naturally released from the cell that are delimited by a lipid bilayer membrane and are incapable of self-replication, i.e., do not contain a functional nucleus.” EVs are a collective name including many subtypes of cell-released, membranous particles, known as microvesicles, microparticles, exosomes, oncosomes, ectosomes, and apoptotic bodies. EVs are characterized by the presence of luminal and transmembrane proteins and attenuation of extracellular or cellular non-EV proteins [141, 142]. The term “exosomes” usually refers to EVs that are formed by the endosomal system, opposite to ectosomes (microparticles and microvesicles) that bud from the plasma membrane. Particularly, intraluminal vesicles are unleashed into the extracellular environment as exosomes when the multivesicular body coalesces with the plasma membrane [143]. Exosomes are identified by their small diameter (40-100 nm) [144]. Moreover, they possess large amounts of tetraspanins (CD81, CD9, and CD63) and annexins, which are commonly used for their characterization [145].

Additionally, exosome vesicles were claimed to possess the ability to induce odontogenesis and augment dental pulp regeneration [146]. Accordingly, a study based on extracted exosome-like vesicles from rat Hertwig's epithelial root sheath (HERS) was tested. Dental pulp cells (DPCs) were united with HERS cell-derived exosome-like vesicles in an in vivo tooth root slice model, triggering the regeneration of hard reparative dentin-like tissue and soft tissue rich in blood vessels and neurons [147]. Moreover, in an interesting study, when SCAP-derived exosomes (SCAP-Exo) were put into a root slice containing BMMSCs and transplanted into immunocompromised mice, dentin and dental pulp-like tissues were formed in the root canal. Besides, when SCAP-Exo were evaluated in vitro, it was reported that dentin sialophosphoprotein expression and hard tissue deposition in BMMSCs treated with SCAP-Exo were significantly upregulated [148]. In another study, EVs were derived from DPSCs and EVs-fibrin gel constructs were manufactured as an in situ delivery system. Afterwards, DPSCs and endothelial cells were cocultured in the constructs. It was reported that EVs-fibrin gels promoted dental pulp regeneration by stimulating collagen deposition and enhancing angiogenesis through upregulating the expression of VEGF [149].

It is further well established that the usage of MSC-derived EVs possesses numerous advantages. First, it overcomes the ethical issues that limit the clinical translation of MSCs. Second, transplanting cells, which might have mutated DNA, can be avoided. Third, the dose of delivered MSCs rapidly declines posttransplant, in contrast to MSC-derived vesicles, which could attain a higher dose. Fourth, EVs are relatively small and can circulate easily, opposite to MSCs, which are too large to circulate smoothly via capillaries. However, the main disadvantage of utilizing MSC-derived vesicles is that they are static and cannot be produced in vivo. Moreover, the efficacy of EVs requires standard parameters to produce EVs of known content, develop storage techniques that preserve vesicle efficacy, and assess their therapeutic potential in well-controlled clinical trials [140].

## 2. Conclusion

Regenerative dentistry is no longer a dream, thanks to the current efforts to imply stem/progenitor cell-based techniques to enhance the regeneration of the pulp-dentin complex and to replace conventional endodontic pulp therapy. Yet, such novel therapies dictate careful testing first in vitro and in animal models, prior to human clinical translation [150]. Cell-based therapies still face many challenges, mainly economical and ethical concerns. Thus, efforts started to target cell homing for pulp-dentin complex regeneration as a simpler, safer, and reasonably priced approach compared to the cell-based transplantation therapy. However, the success and safety of MSCs administered via IV or IA routs, as well as directing such cells towards the injured tissues, are not always guaranteed. Despite the great advancements in pulp-dentin complex regeneration through cell homing in the past years, they require further investigations and development. Cell homing techniques need to be examined in more realistic models, starting with animals then humans. Moreover, clinical trials are crucial to point out possible indications and contraindications. Thus, numerous aspects still need to be resolved to make it applicable and with predictable outcomes in clinical dental practice. The perspective of replacing conventional endodontic therapy, while retaining the tooth vitality in a practical and relatively safe way, provides hope for the clinical dental practice. Finally, any minor step towards the future is counted as an additional profit that must be preciously handled and searched thoroughly to be utilized later in the field of regenerative dentistry.

## Figures and Tables

**Figure 1:**
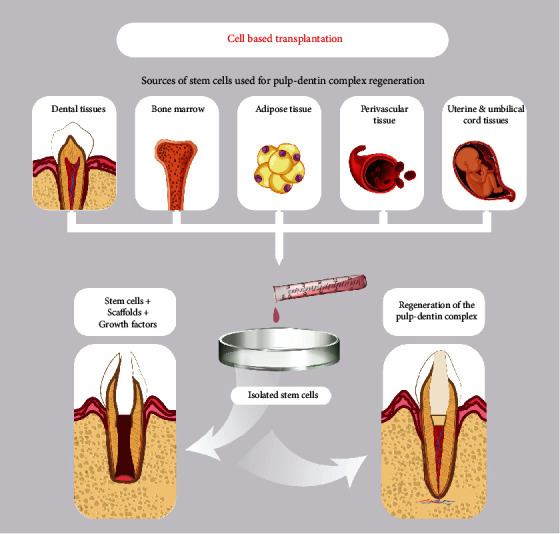
Cell-based transplantation method and sources of stem cells used for pulp-dentin complex regeneration.

**Figure 2:**
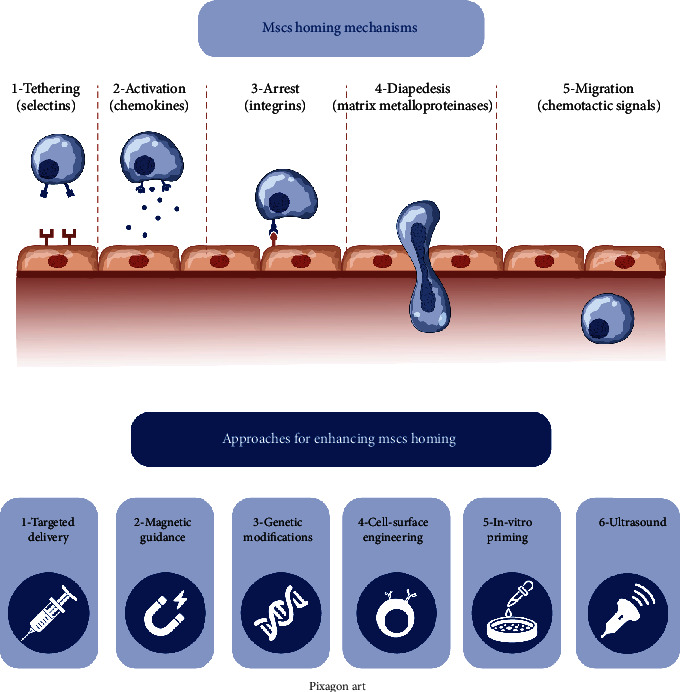
MSC homing mechanisms and different approaches for enhancing MSC homing.

**Figure 3:**
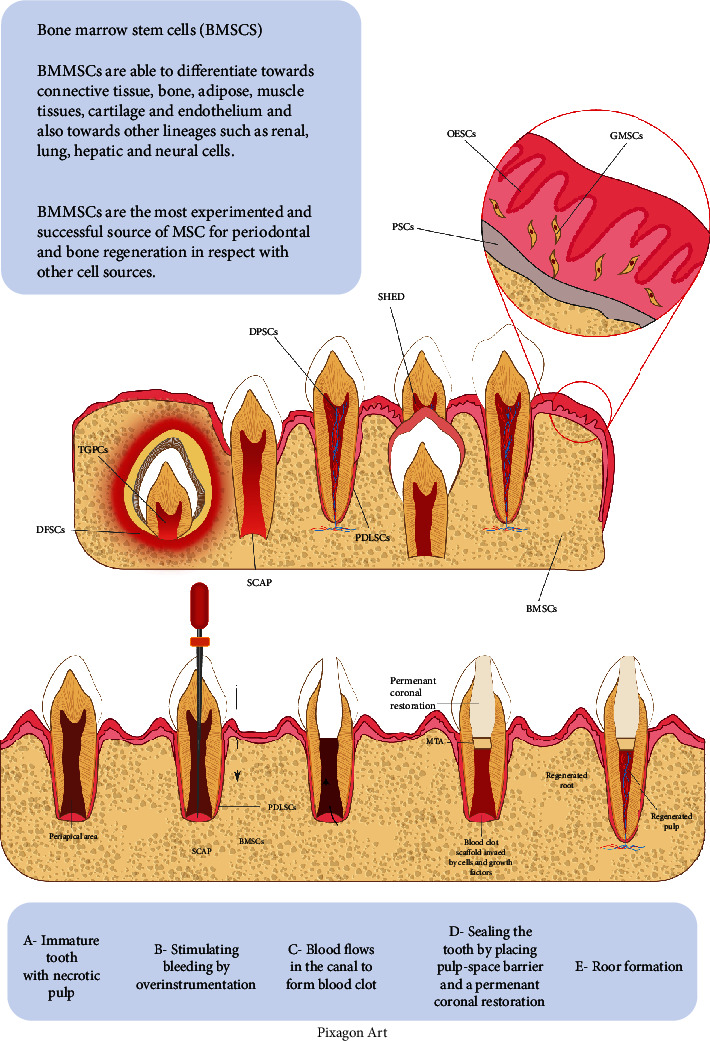
Different sources of stem/progenitor cells in the oral cavity and steps of revascularization.

**Table 1:** Summary of cell-based transplantation studies for pulp-dentin complex regeneration.

Study	Study design				Outcomes		
	Blind; random; design	Animal model/human	Type of study	Groups	Primary outcomes	Secondary outcomes	Histology
					Clinically and radiographically	Discoloration and sensibility test	
Chen et al. 2015	Randomized controlled trial	Animal study; 3 dogs providing 60 root canals	Cell based	Group I: DPSC/PRF construct Group II: DPSCs only Group III: PRF granules only Group IV: blank controls without any exogenous transplanted grafts			DPSC/PRF construct led induced regeneration of dense pulp-like tissues with richly distributed blood capillaries. The deposition of regenerated dentin alongside the intracanal walls was evident.
Cai et al. 2016	Randomized controlled trial	Animal study; 6 rats, 12 incisors	Cell based	Group I: 6 untouched incisors Group II: 2 sham incisors Group III: 4 transplanted incisors			Immunohistochemistry revealed globular dentin and pulp-like tissue formation.
Jia et al. 2016	Randomized controlled trial	Animal study; 18 immature premolars from 2 dogs	Cell based	Group I: mineral trioxide aggregate Group II: absorbable gelatin sponge Group III: cDPSCs Group IV: Simvastatin group	Simvastatin stimulated cDPSC mineralization and induced DPSC pulp and dentin regeneration.	After 10 weeks, radiographic examination of pulpotomized teeth showed closure of the root apex and thickening of the root canal wall.	cDPSCs pretreated with SI, regenerated pulp that was filling most of the pulp cavity. Newly formed dentin deposits succeed to the primary dentin and odontoblasts were evident in the regenerated area. It was significantly higher than that in all other groups.
Iohara et al. 2016	Randomized controlled trial	Animal study; a total of 28 teeth from 5 dogs were randomly divided into 4 groups.	Cell based	Group I: pulpectomy only (no cells and no collagen) Group II: normal teeth Group III: transplantation of MDPSCs and 7.5 ng/mL G-CSF with an atelocollagen scaffold Group IV: collagen only	The signal intensity (SI) of MRI of the normal teeth was significantly higher than that of nonvital pulpectomized teeth and the controls of collagen transplanted teeth at 90 days. The stem cell transplanted teeth showed gradual decrease in the SI until 180 days which was similar to the normal teeth and significantly higher than that in the teeth transplanted with collagen alone without the stem cells.		One day after transplantation of collagen alone or MDPSCs and G-CSF with collagen, the root canal was filled with collagen like-fibers. Ninety days after the transplantation of MDPSCs and G-CSF with collagen, most of the root canal was filled with pulp-like tissue in which well-developed vasculature and dentin were formed along the dentinal wall. On day 180, the root canal was completely filled with pulp-like tissue and secondary dentin was formed in the apical part and along the dentinal wall.
Bakhtiar et al. 2017	Randomized controlled trial	Animal study; 32 premolars of 5 dogs	Cell based	Group A: MTA Group B: TDM Group C: TCP Group D: TDM scaffold impregnated with DPSCs+TDM Group E: TCP scaffold impregnated with DPSCs+TCP	The negative control group showed severe inflammation and granulation tissue formation. The positive control group was characterized by intact periodontal tissues and no inflammation.		Dentin bridge formation was absent in specimens of all groups. The SC+TDM group was associated with significantly more bone formation than other groups. Cementum was formed with a cellular and continuous pattern in all specimens.
Ito et al. 2017	Randomized controlled trial	Animal study; 48 female Wistar rats	Cell based	Group 1: RBMMSC/PLLA/Matrigel constructs Group 2: Matrigel constructs without RBMMSC Constructs were implanted into the cavity for 3, 7, or 14 days (*n* = 8 in each group).	Immunohistochemistry revealed that nestin-expressing odontoblast-like cells beneath the dentin at the border of implanted area increased until 14 days.		Considering RBMMSC constructs at 3 days, cells were located mainly along the implanted scaffolds. At 7 days, pulp tissue regeneration was created in almost the entire implanted region. At 14 days, pulp tissue regeneration continued throughout the implanted region.
Mangione et al. 2017	Randomized controlled study, split-mouth model	Animal study; 3 minipigs, of total 48 teeth	Cell based	Group 1: pDPCs were implanted in the left maxillary and mandibular teeth. Group 2: no pDPC scaffold was implanted in teeth of the right side.	Micro-CT examination of the treated teeth showed the formation of a reparative mineralized bridge in the remaining pulp of both groups. External root resorption was evident in all teeth.		With pDPSCs, reparative dentin bridge presented many abundant and joined nonmineralized areas.
Sueyama et al. 2017	Randomized controlled trial	Animal study; 40 female rats	Cell based	Group 1: PLLA implanted scaffolds with MSCs and Ecs Group 2: implanted scaffolds with MSCs Group 3: implanted acellular scaffolds Group 4: pulpotomy cavities were sealed with MTA only. Group 5: no pulpotomy (used as the normal control)	14 days after implantation; MSCs associating Ecs accelerated the pulp tissue regeneration and enhanced dentin bridge formation.		Teeth with MSC/EC constructs showed pulp healing and complete dentin bridge formation, but MSCs alone showed incomplete, thinner dentin bridges. Teeth implanted with acellular scaffolds were of poor tissue regeneration in the implanted area and incomplete hard tissue formation. Teeth subjected to pulpotomy without implantation did not show pulp tissue regeneration.
El Ashiry et al. 2018	Randomized controlled trial, split-mouth design	Animal study; 12 dogs, 36 teeth	Cell based	Group A: tooth transplanted with a construct of autologous dental pulp stem cells with growth factors seeded in a chitosan hydrogel scaffold Group B: tooth received only growth factors with scaffold.	DPSC constructs resulted in complete root maturation., radicular thickening, root lengthening, and apical closure.		DPSC constructs showed regeneration of pulp-dentin-like tissue filling the emptied canals. The vascularized pulp-like tissue resembled the natural pulp. On the contrary, in the other group, no soft tissues were observed.
Cordero et al. 2020	Case report	Human mature molar with accidental root perforation	Cell based		Radiographic and cone-beam computed tomographic images indicated remission of the apical lesion. Clinically, normal responses to percussion and palpation tests	Tooth was responsive to the electric pulp test, and the vitality test indicated low blood perfusion units.	
Iohara et al. 2020	Randomized controlled trial	Animal study; aged dogs	Cell based	Group I: no treatment Group II: nanobubble treatment Group III: 0.05% trypsin for 10 min Group IV: 0.5% trypsin for 10 min Group V: 0.05% trypsin for 30 min Group VI: 0.05% trypsin for 10 min with nanobubbles			The amount of pulp-like regenerated tissues was three-times higher with 0.05 and 0.5% trypsin pretreatment for 10 min than that in the no treatment group. Moreover, the trypsin pretreatment induced higher pulp tissue vascularization compared with no pretreatment.

**Table 2:** Summary of cell homing studies for pulp-dentin complex regeneration.

Study	Study design				Outcomes		
	Blind; random; design	Animal model/human	Type of study	Groups	Primary outcomes	Secondary outcomes	Histology
					Clinically and radiographically	Discoloration and sensibility test	
Thibodeau et al. 2007	Randomized clinical study	Animal study; 60 immature teeth from 6 dogs	Cell homing	Group 1: no treatment (but disinfected) Group 2: blood clot Group 3: collagen solution Group 4: collagen solution+blood clot Group 5: negative control (left untouched)	Radiographic thickening of root canal walls, apical closure, and healing of periapical radiolucency in all the groups		Hard tissue deposition on radicular dentin in all groups except the negative control New vital tissues were formed in the root canals in all groups except in the negative group.
Shah et al. 2008	Pilot clinical study	14 cases of infected immature teeth	Cell homing	Blood clot revascularization	Radiographic resolution of periapical radiolucencies was judged to be good to excellent in 93% of the cases. The striking finding was complete resolution of clinical signs and symptoms and appreciable healing of periapical lesions in 78% of cases.		
Ding et al. 2009	Clinical study	12 patients, each with immature permanent tooth with chronic or acute apical periodontitis	Cell homing	Blood clot revascularization	Teeth (*n* = 3) were found to exhibit complete root development with a positive response to pulp testing.		
Lovelace et al. 2011	Clinical study	A total of 12 patients were included in this study.	Cell homing	This study consisted of 6 boys and 6 girls with immature permanent maxillary or mandibular single rooted immature tooth with open apices with diagnosis of pulp necrosis with apical periodontitis.	Molecular analyses of blood collected from the canal system indicated the significant accumulation of transcripts for stem cell markers CD73 and CD105 (up to 600-fold).		Histological analysis demonstrated that the delivered cells expressed both CD105 and STRO-1, markers for a subpopulation of mesenchymal stem cells.
Jadhav et al. 2012	Pilot clinical study	20 patients with nonvital, immature anterior teeth were randomly categorized into 2 groups; revascularization with or without PRP	Cell homing	Group I: blood clot Group II: using PRP	Clinically, all cases were asymptomatic with complete resolution of signs and symptoms. Radiographically, there was a marked difference in periapical healing, apical closure, and dentinal wall thickening in group II in comparison with group I; however, root lengthening was comparable for both of the procedures.		
Shimizu et al. 2012	Case report	Human study	Cell homing	Revascularization/regeneration procedure			At 3.5 weeks after revascularization, more than one half of the canal was filled with loose connective tissue similar to the pulp tissue. A layer of flattened odontoblast-like cells lined along the predentin. Layers of epithelial-like cells, similar to the Hertwig's epithelial root sheath, surrounded the root apex. No hard tissue was formed in the canal.
Mishra et al. 2013	Case report	An 11-year-old boy with the history of trauma was diagnosed with pulpal necrosis and symptomatic apical periodontitis in tooth #21.	Cell homing	Platelet-rich fibrin used	Clinical examination at 6 and 12 months revealed no sensitivity to percussion and palpation in tooth #21, and it responded positively to both electric pulp and cold tests. Radiographic examination showed resolution of periapical rarefaction, further root development and apical closure of the tooth #21 and its associated supernumerary tooth.		
Zhang et al. 2014	Randomized clinical study	Animal study; three 6-month-old beagles	Cell homing	Group 1: PRP Group 2: blood clot Group 3: negative control			Apical apex was closed. Pulp-like tissue (fibroblasts and blood vessels) was developed. Thickening of the canal wall with ingrowth of cellular cementum-like tissues (cementocyte-like cells) were present in the newly formed tissues. Large number of inflammatory cells were present in the PRP and blood clot groups.
Priya et al. 2015	Clinical case study	The present case evaluated PRP for pulpal regeneration in an avulsed mature incisor (>8-hour extraoral dry time) of an 11-year-old boy after delayed replantation.	Cell homing	The present case evaluated PRP for pulpal regeneration in an avulsed mature incisor (>8-hour extraoral dry time) of an 11-year-old boy after delayed replantation.	Nine- and 12-month radiographs revealed resolution of periapical radiolucency with no further progression of internal resorption. The tooth showed a positive response to thermal and electric pulp tests. The findings observed in this case warrant further research under controlled conditions to evaluate endodontic and periodontal regeneration in a tooth that would otherwise be expected to have an unfavourable prognosis.		
El Ashiry et al. 2016	Clinical study	20 patients with immature necrotic teeth with apical periodontitis	Cell homing	Blood clot group	Within 12-24 months, increase in dentinal wall thickness and root length and continued root development were observed.		
Shivashankar et al. 2017	Triple-blind randomized clinical trial	60 patients with necrotic immature tooth	Cell homing	Group A: PRF (scaffold) Group B: revascularization with conventional induced bleeding Group C: PRP (biomaterial)	At the end of 12 months, patients presented no pain and no signs of reinfection. No radiographic enlargement of the preexisting apical pathosis in all the three groups		
Song et al. 2017	Retrospective study	29 cases undergone revascularization between 2010 and 2014.	Cell homing	Revascularization group	Continued root development with apical closure in 79.35 of cases Revascularization associated intracanal calcification in 62.1% of the cases after 12-month follow-up		
Nageh et al. 2018	Clinical study	15 patients with necrotic pulp with symptomatic or asymptomatic apical periodontitis	Cell homing	PRF revascularization	All teeth survived after 12 months, no pain or swelling.	Pulp sensibility regained using electric pulp tester in 9 cases after 12-month follow-up.	
Neelamurthy et al. 2018	Clinical study	15 patients with immature and mature permanent teeth with pulpal necrosis and open apices	Cell homing	Bleeding group	After 10 months, 10 out of 13 patients showed root development and apical closure.	2 out of 13 patients showed a positive response to electric sensibility test.	
Arslan et al. 2019	Randomized clinical study	56 mature necrotic teeth with large periapical radiolucency	Cell homing	Group I: conventional root canal treatment (CRCT) Group II: regenerative endodontic procedures (REP)	No difference between the two groups regarding pain, palpation, swelling, sinus tract, and pain on percussion. Radiologically, absence and reduction of the radiolucency were 85% in the CRCT group and 92.4% in the REP group.	50% of REP-treated teeth responded positively to electrical vitality testing.	
Mittal et al. 2019	Clinical study	16 cases of necrotic immature permanent teeth using PRF, collagen, Placentrex, and chitosan	Cell homing	Group I: PRF Group II: collagen Group III: Placentrex Group IV: chitosan	Clinically, patients were completely asymptomatic throughout the study period. Radiographically, all cases showed an improvement in terms of periapical healing, apical closure, root lengthening, and dentinal wall thickening. PRF and collagen gave better results than Placentrex and chitosan in terms of periapical healing, apical closure, and dentinal wall thickening.		
Ragab et al. 2019	Randomized controlled trial	22 patients suffering from immature necrotic permanent maxillary central incisors	Cell homing	Group A: blood clot Group B: using PRF revascularization	After a follow-up period of 12 months, most of the cases showed radiographic evidence of periapical healing and showed calcific bridges either cervical and/or apical.		
Arora et al. 2020	Case series	9 patients with infected immature molars	Cell homing	Bleeding group	After 60 months, all teeth showed continued root development and maintained functionality.	None responded to vitality testing.	
Elsheshtawy et al. 2020	Randomized controlled trial	26 patients with immature permanent anterior teeth with necrotic pulps	Cell homing	Group 1: PRP (test) Group 2: blood clot (control)	All cases in both groups showed complete healing after 3 months. One tooth in the PRP group had signs of reinfection after 6 months. In both groups, there was increase in root lengths and dentinal root widths and decrease in the apical foramen width and periapical area diameter.	No change in pulp sensibility using thermal and electrical pulp testing	
Rizk et al. 2020	Double-blinded randomized controlled trial	26 patients with maxillary permanent immature central incisors	Cell homing	Group I: PRP (scaffold) Group II: PRF (scaffold)	All teeth were survived after 12 months. Both groups showed marginal increase in radiographic root length and width. Increase in periapical bone density Decrease in apical diameter		
Rizk et al. 2020	Split-mouth double-blind randomized controlled trial	15 patients with bilateral necrotic upper permanent central incisors with open apex	Cell homing	Group I: blood clot Group II: PRF	Apical diameter in the PRF group is greater than of the blood clot group.		

## Data Availability

Data are available on request.

## Abbreviations

AAE: American Association of Endodontists
BMMSCs: Bone marrow mesenchymal stem cells
BMPs: Bone morphogenetic proteins
BMP-2: Bone morphogenic protein-2
CCR2: C-C chemokine receptor type 2
CCR3: C-C chemokine receptor type 3
CCR4: C-C chemokine receptor type 4
CD105: Cluster of differentiation 105
CD44: Cluster of differentiation 44
CD49d (*α*4*β*1): Integrin *α*4
CD73: Cluster of differentiation 73
cDPSCs: Canine dental pulp stem cells
CRCT: Conventional root canal treatment
CX3CR1: CX3 chemokine receptor 1
CXCR4: C-X-C chemokine receptor type 4
CXCR7: C-X-C chemokine receptor type 7
DPCs: Dental pulp cells
DPSCs: Dental pulp stem cells
EB: Evoked bleeding
ECs: Endothelial cells
EDTA: Ethylenediaminetetraacetic acid
EGF: Epidermal growth factor
FGF: Fibroblast growth factor
G-CSF: Granulocyte colony-stimulating factor
GSK-3: Glycogen synthase kinase
HCELL: Hematopoietic cell E-/L-selectin ligand
hDPSCs: Human dental pulp stem cells
HERS: Hertwig's epithelial root sheath
HIF-1a: Hypoxia-inducible factor-1a
IA: Intra-arterial
IGF-1: Insulin-like growth factor-1
IL-1*α*: Interleukin-1 alpha
IL-1*β*: Interleukin-1 beta
IL-4: Interleukin-4
IL-6: Interleukin-6
IL-8: Interleukin-8
IL-10: Interleukin-10
IV: Intravenous
MDPSCs: Mobilized dental pulp stem cells
MMP-1: Matrix metalloproteinase-1
MMPs: Matrix metalloproteinases
MRI: Magnetic resonance imaging
MSCs: Mesenchymal stem/progenitor cells
MTA: Mineral trioxide aggregate
PDGF: Platelet-derived growth factor
pDPSCs: Pocrine dental pulp stem cells
PLLA: Poly L-lactic acid
PPP: Platelet-poor plasma
PRF: Platelet-rich fibrin
PRP: Platelet-rich plasma
RBMMSC: Rat bone marrow mesenchymal stem cells
REP: Regenerative endodontic procedures
SC: Stem cell
SCAP: Stem cells of apical papilla
SDF-1: Stromal cell-derived factor
SHED: Stem cells from human exfoliated deciduous teeth
SI: Signal intensity
SIM: Simvastatin
STRO-1: Stromal cell surface marker-1
TCP: Tricalcium phosphate
TDM: Treated dentin matrix
TGF*β*1: Transforming growth factor beta 1
TNF-*α*: Tumor necrosis factor
UCMSCs: Umbilical cord mesenchymal stem cells
VCAM-1 (CD106): Vascular cell adhesion molecule 1
VEGF: Vascular endothelial growth factor
VLA-4: Integrin VLA-4.
